# 1430. Outbreak Clusters of Invasive Group A *Streptococcus* Associated with Wound Care in Long-term Care Facilities

**DOI:** 10.1093/ofid/ofad500.1267

**Published:** 2023-11-27

**Authors:** Jennifer Zipprich, Kathryn Como-Sabetti, Kayla M Bilski, Paula S Vagnone, Jennifer L Dale, Xiong Wang, Sopio Chochua, Bernard Beall, Nicholas Lehnertz, Sarah Lim, Kristi K Juaire, Tammy Hale, Ruth Lynfield

**Affiliations:** Minnesota Department of Health, St. Paul, Minnesota; Minnesota Department of Health, St. Paul, Minnesota; Minnesota Department of Health, St. Paul, Minnesota; MN EIP, Minneapolis, Minnesota; Minnesota Department of Health Laboratory, St. Paul, Minnesota; Minnesota Department of Health, St. Paul, Minnesota; CDC, Atlanta, Georgia; Centers for Disease Control and Prevention/Retired, Atlanta, Georgia; Minnesota Department of Health, St. Paul, Minnesota; Minnesota Department of Health, St. Paul, Minnesota; Minnesota Department of Health, St. Paul, Minnesota; Minnesota Department of Health, St. Paul, Minnesota; Minnesota Department of Health, St. Paul, Minnesota

## Abstract

**Background:**

Residents of long-term care facilities (LTCFs) are at increased risk of invasive group A *Streptococcus* (iGAS) infections. It is important to identify intrafacility and interfacility iGAS transmission clusters.

**Methods:**

Minnesota Department of Health (MDH) conducts active surveillance for iGAS infections through the Emerging Infections Program, including necrotizing fasciitis, bacteremia, and other normally sterile sites. Clinical and demographic information, including residence, is collected from medical records, and isolates are submitted to MDH and the Centers for Disease Control and Prevention for characterization. Whole genome sequencing (WGS) was used to determine *emm* type and identify clusters (isolates exhibiting ≤15 single nucleotide polymorphisms [SNPs]). In 2016–18 and 2022–23, iGAS clusters were investigated among LTCF residents.

**Results:**

From March 2016 to May 2018, 749 iGAS cases were reported, 10% were among LTCF residents. Forty iGAS *emm*89 case-isolates (pairwise distance of 0–11 SNPs, recovered from LTCF residents at 19 different LTCFs, 1–7 cases/LTCF) defined cluster A. From January 2022–March 2023, 489 iGAS cases were reported, 7% were LTCF residents. Twenty iGAS cases (0–7 SNPs, recovered from LTCF residents at 10 different LTCFs, 1–8 cases/LTCF) defined cluster B. The two clusters represented multiple infection types including bacteremia (40%), septic shock (11%), pneumonia (8%), cellulitis with bacteremia (29%), and one case of necrotizing fasciitis. All were hospitalized and 23% died; 76% had wound care prior to iGAS infection. Among cluster A cases 58% had care from a single wound care physician, and among cluster B cases 36% received wound care from a single (but different) wound care physician. Identification of clusters led to MDH investigation on site.

**Conclusion:**

iGAS is associated with severe morbidity and mortality among LTCF residents. We identified two iGAS clusters, each with cases among multiple LTCFs. Investigations identified common wound care providers and lapses in infection prevention practices. WGS enabled identification of complex multi-facility iGAS clusters, additional epidemiologic links, and development of facility-specific recommendations.

**Disclosures:**

**Jennifer Zipprich, PhD**, Pfizer: Spouse employment

